# On the Hydration State of Amino Acids and Their Derivatives at Different Ionization States: A Comparative Multinuclear NMR and Crystallographic Investigation

**DOI:** 10.1155/2012/565404

**Published:** 2012-05-14

**Authors:** Charalampos G. Pappas, Andreas G. Tzakos, Ioannis P. Gerothanassis

**Affiliations:** Section of Organic Chemistry and Biochemistry, Department of Chemistry, University of Ioannina, 45110 Ioannina, Greece

## Abstract

^2^D, ^13^C, ^14^N, and ^17^O NMR and crystallographic data from the literature were critically evaluated in order to provide a coherent hydration model of amino acids and selected derivatives at different ionization states. ^17^O shielding variations, longitudinal relaxation times (*T*_1_) of ^2^D and ^13^C and line widths (Δ*ν*_1/2_) of ^14^N and ^17^O, may be interpreted with the hypothesis that the cationic form of amino acids is more hydrated by 1 to 3 molecules of water than the zwitterionic form. Similar behaviour was also observed for N-acetylated derivatives of amino acids. An exhaustive search in crystal structure databases demonstrates the importance of six-membered hydrogen-bonded conjugated rings of both oxygens of the *α*-carboxylate group with a molecule of water in the vicinity. This type of hydrogen bond mode is absent in the case of the carboxylic groups. Moreover, a considerable number of structures was identified with the propensity to form intramolecular hydrogen bond both in the carboxylic acid (NH*⋯*O=C) and in the carboxylate (NH ⋯ O^−^) ionization state. In the presence of bound molecules of water this interaction is significantly reduced in the case of the carboxylate group whereas it is statistically negligible in the carboxylic group.

## 1. Introduction

Water plays a fundamental role in the conformation and activity of every biological macromolecule [1]. Peptide- and protein- hydration is the dominant factor in the stabilization of spatial molecular structure, in the process of protein folding by gating hydrophobic residues, and in the mechanisms of peptide and protein mediated reactions [1–4]. Water molecules, therefore, can be considered as an integral component of biomolecular systems with dynamic, functional, and structural roles [4–7]. Investigation of the structural and functional role of water molecules, bound to proteins and peptides, requires a sufficient understanding of the hydration process of their building blocks [1, 2]. The hydration of amino acids and their derivatives at a molecular level, therefore, is of great importance and has been extensively studied with X-ray crystallography [1, 3] and a variety of spectroscopic techniques including multinuclear magnetic resonance spectroscopy [2, 8–13], IR and Raman spectroscopy [14–16], ICR mass spectrometry [17], and laser ablation in combination with microwave spectroscopy [18].

We present here, for the first time in the international literature, a comparative investigation of literature ^2^D, ^13^C, ^14^N, and ^17^O NMR and crystallographic data in order to provide a coherent hydration model of amino acids and selected derivatives at different ionization states in aqueous solution and in the crystal state.

## 2. Results and Discussion

### 2.1. ^17^O NMR Shieldings

**Figure 1:**
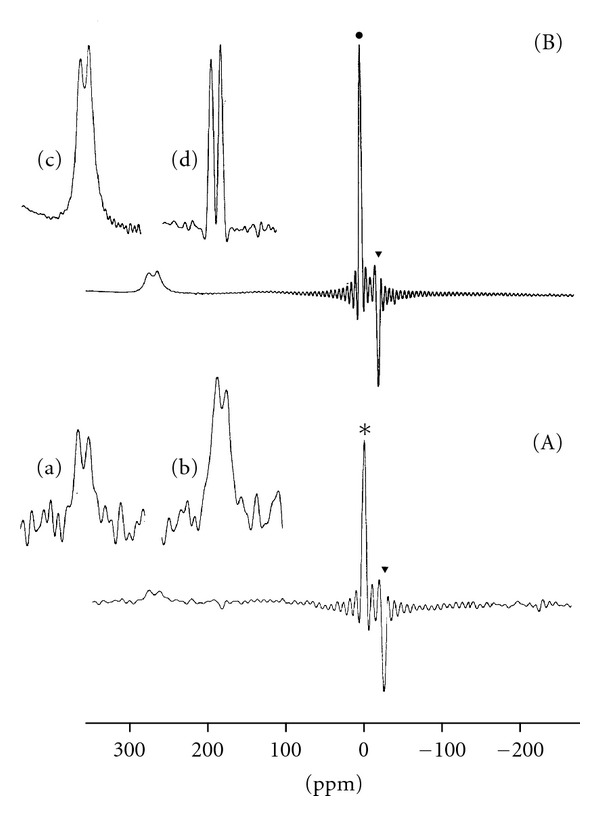
27.11 MHz ^17^O NMR spectra of 0.1 M solutions of glutamic acid in H_2_O containing 1 M NaCl, temperature = 40°C, pH = 3.1. (a) Natural abundance spectrum in ^17^O depleted water (*). *T*_acq_ = 5 ms, NS = 3 × 10^6^, total experimental time ca.4.2 h; *▼* transmitter's residual peak. Glutamic acid resonances after (a) vertical expansion (8x); (b) exponential multiplication of the FID (LB = 100 Hz). (b) Spectrum of 1% enriched glutamic acid in ordinary water (●). *T*_acq_ = 7.5 ms, NS = 150,000, total experimental time ca. 19 min. Glutamic acid resonances after (c) vertical expansion (8x); (d) multiplication of the FID with a Gaussian-exponential function. (Reprinted, with permission, from *Helv. Chim. Acta* [23]. Copyright 1982, by Swiss Chemical Society).

^17^O NMR has received little attention in amino acid and peptide research [2, 12, 13, 19, 20]. This neglect is due to the fact that of the three naturally occurring oxygen isotopes, only ^17^O possesses a nuclear spin (*I* = 5/2). Owing to its electric quadrupole moment (Qe = −2.6 × 10^−30^ em^2^) and, thus, broad line widths, and its low absolute sensitivity compared with that of ^1^H (~1.1 × 10^−5^), the ^17^O- isotope is one of the more difficult to observe by NMR spectroscopy [12, 13, 21, 22]. ^17^O NMR studies, therefore, of compounds at natural abundance require high concentrations (>0.1 M) and extensive signal averaging. Recording of spectra can be greatly facilitated by the use of ^17^O enriched samples [23–27].  Figure 1(a) illustrates the natural abundance ^17^O NMR spectrum of glutamic acid, 0.1 M in ^17^O-depleted water at 40°C. Despite the extensive signal averaging (number of scans (NS) =3 × 10^6^) and the total experimental time of 4.2 hours, the achievable signal-to-noise (*S*/*N*) ratio is very poor and practically prohibitive for the accurate determination of chemical shifts and line widths.  Figure 1(b) illustrates the clear advantages of working with ^17^O-labelled glutamic acid (^17^O enrichment 1 at.%) [23].

^17^O shieldings of various chemical functional groups are very sensitive for studying hydrogen bonding interactions because of the large chemical shift range of the ^17^O nucleus [12, 13]. The effect of solvent-induced hydrogen bonding interactions on *δ*(^17^O) of the carboxyl groups is, however, rather small compared with the substantial sensitivity of over 80 ppm to hydrogen bonding interaction of *δ*(^17^O) of amide and carbonyl oxygens [12, 13]. Only a single ^17^O resonance absorption is observed for the carboxylic group since the shifts of the individual resonance absorptions *δ*(C=O) and *δ*(OH) are averaged out by rapid intermolecular proton transfer with protic solvents, traces of H_2_O, and/or through hydrogen bonding aggregates of the COOH groups in organic solvents [12, 13, 23, 24, 26, 28]. Reuben [29] from dilution studies of acetic acid in 1,2-dichloroethane estimated a deshielding effect of ~12 ppm due to breaking of a hydrogen bond involving the carbonyl oxygen of the acid and a shielding effect of −6 ppm due to breaking of a OH*⋯*O hydrogen bond. Therefore, a total shift of only +6 ppm is expected for the monomeric acetic acid in apolar media (dichloroethane) compared with the dimeric form.

**Figure 2:**
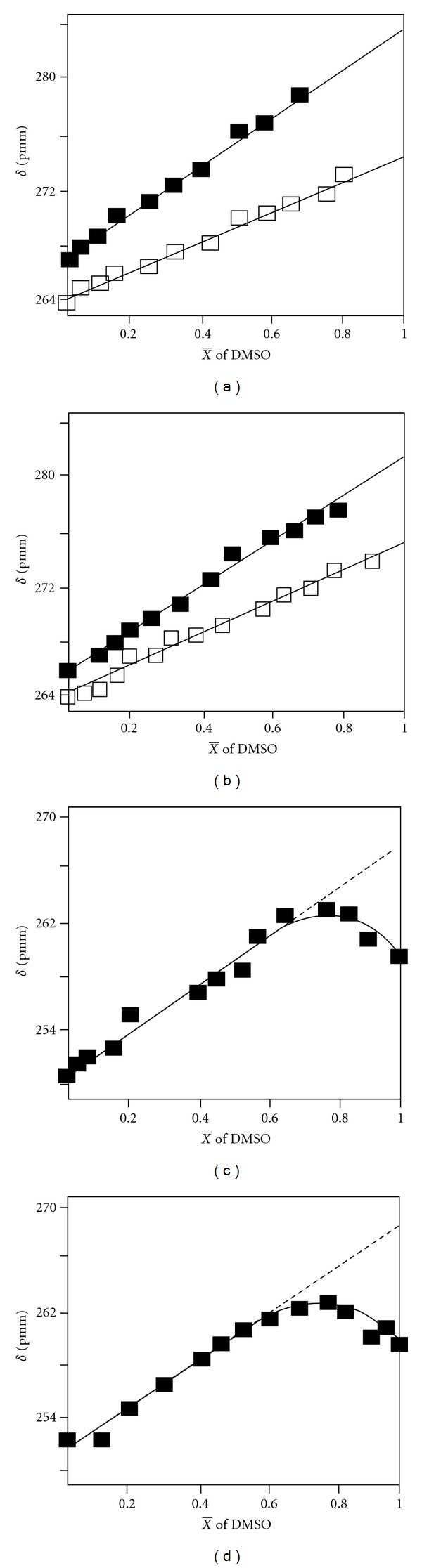
Dependence of the ^17^O chemical shift (ppm from H_2_O) of L-alanine and L-proline on the molar fraction X of DMSO at various pH values. (a) L-alanine at pH 7.4 (■) and pH 11.9 (□), (b) L-proline at pH 8.3 (■) and pH 13.0 (□), (c) L-alanine at pH 1.5, and (d) L-proline at pH 1.0. (Adapted, with permission, from *Biochem. Biophys. Res. Commun.* [9]. Copyright 1986, Academic Press, Inc.).

Despite the relatively low sensitivity of the ^17^O shieldings of the carboxyl group to hydrogen bond interactions, Spisni and collaborators [9] attempted to estimate the solvation state of the *α*-carboxyl group of amino acids in the different ionization states. Figures 2(a) and 2(b) show the dependence of *δ*(^17^O) of L-alanine and L-proline as a function of molar fraction of DMSO in the pH range 7-8 and 12-13. Since DMSO cannot form a hydrogen bond interaction with the carboxylate group, contrary to the case of H_2_O, the shielding difference of 10–17 ppm between the two solvents was interpreted with the hypothesis that the carboxylate group of these amino acids is hydrated by two water molecules in aqueous solution with one hydrogen bond per carboxylate oxygen. In the acidic pH range (Figures 2(c), 2(d)), a nonlinear behaviour of the chemical shift at high DMSO molar fractions was observed. For DMSO molar fractions up to 0.6, a linear dependence of the chemical shift was observed which, on extrapolation to 100% DMSO, results in a shielding of 15–17 ppm, the same as in the neutral pH. This was interpreted with the hypothesis that two hydrogen bonds (one to each oxygen) are being ruptured. When the DMSO molar fraction is between 0.6 and 0.8, it was suggested that a third molecule of water, which is hydrogen bonded to the hydroxyl hydrogen, is dissociated due to the interaction with DMSO. This might explain the deflection from linearity and the plateau-like dependence of the ^17^O shielding. The protonated form, therefore, of the carboxyl group of the amino acids is more hydrated with an access of a bound molecule of H_2_O than the deprotonated form. This conclusion is in qualitative agreement with multinuclear NMR relaxation data (see below).

### 2.2. Multinuclear NMR Relaxation Data

For quadrupolar nuclei, such as ^2^D, ^15^N, and ^17^O, the longitudinal (*T*_1_) and transverse (*T*_2_) relaxation times are essentially due to quadrupolar interaction

(1) $$\frac{1}{T_{1}}=\frac{1}{T_{2}}\left(1+\frac{\eta^{2}}{3}\right)\chi^{2}f\left(\omega ,D\right),$$

where *χ* is the nuclear quadrupole coupling constant. The asymmetry parameter *η* varies from 0 to 1 and describes the deviation of the electric field gradient from axial symmetry, and *f*(*ω*, *D*) is the correlation function, which depends on the rotational diffusion constant *D* and its relative orientation with respect to the principal axes of the field gradient tensor [12, 13]. When isotropic reorientation is assumed, *f*(*ω*, *D*) reduces to a single overall correlation time *τ*_mol_ which is given by the Stokes-Debye formula

(2a) $$\tau_{\text{mol}}=\frac{V_{m}\eta_{\nu }}{k_{B}T},$$

where *V*_*m*_ is the molecular volume, *η*_*ν*_ the viscosity of the solution, *k*_*B*_ the Boltzman constant, and *T* the absolute temperature. *V*_*m*_ can be estimated as

(2b) $$V_{m}=\frac{0.74\text{MW}}{N_{0}\rho },$$

where *N*_0_ is the Avogadro's number and MW and *ρ* are the molecular weight and the density of the solute (amino acid), respectively.

**Figure 3:**
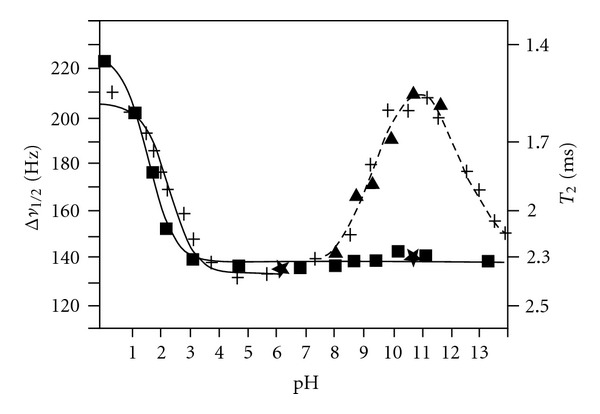
The pH-dependence of the ^17^O NMR linewidth of 0.1 M glycine in H_2_O which contained 1 M NaCl; temperature 40°C; (+) measured at 27.11 MHz; (▲) at 12.2 MHz; (■) at 27.11 MHz after addition of 2 mM EDTA (ethylenediamine-N,N,N,N-tetraacetate). The dashed lines were drawn to follow the experimental points. The solid lines correspond to a nonlinear least-squares fit to one proton titration curve. (★) are *T*_1_ values (ms) measured at 27.11 MHz. (Reprinted, with permission, from *Helv. Chim. Acta* [24]. Copyright 1982, by Swiss Chemical Society.).

Paramagnetic impurities shorten the relaxation times and might lead to erroneous results. The removal, therefore, of these impurities is necessary in studies of *T*_2_ and *T*_1_ relaxation times. Figure 3 illustrates the pH dependence of the ^17^O line widths of 0.1 M glycine in H_2_O [24]. A broad minimum between pH 4 and 7 was observed. This ^17^O line width minimum has been previously explained by a decrease of the molecular tumbling time attributable to a reduction in hydration and, thus, intermolecular association of glycine in the zwitterionic form [26]. In the high pH region, a broad maximum at pH ≈11 was observed. Addition of 2 mM ethylenediamine-N,N,N′,N′-tetraacetate (EDTA) to the original solution resulted in no line width variation in the neutral and high pH region. It can, therefore, be concluded that this broad minimum at pH ≈11 should be attributed to the effect of paramagnetic impurities and not to a hydration change of glycine in the neutral and high pH region [24]. 

^2^D *T*_1_ relaxation times of C_*α*_D_2_ of glycine at acidic pH were shown to be shorter relative to those at neutral pH [8]. This shortening in *T*_1_ implies an increase in *τ*_mol_ and, thus, in the effective molecular weight MW ((1), (2a), and (2b)), which was interpreted with an increase in the hydration state in the cationic form.

Tritt Goc and Fiat [30] investigated in detail the viscosity and temperature dependence of the ^17^O NMR line width of glycine, alanine, proline, leucine, histidine, and phenylalanine at pH 2, 7, and 12.5. The experimentally observed viscosity/temperature (*η*_*ν*_/*T*) dependence of the reorientation correlation time was compared with various hydrodynamic models. A model of the hydration state in the primary solvation sphere of the carboxylic group of amino acids in their cationic state was suggested in which two water molecules are hydrogen bonded to the oxygens and one to the hydrogen of the OH group. In the zwitterionic and anionic states, the hydration model of the carboxylate group can be presented by a structure in which one water molecule is hydrogen bonded to each of the oxygens [30].

The ^17^O [10, 11] and ^14 ^N NMR [11] line widths of several protein amino acids were measured in aqueous solution to investigate the effect of molecular weight on the line widths (Table 1). The ^14 ^N and ^17^O line widths, under composite proton decoupling, increase with the bulk of the amino acid, and increase at low pH. Assuming an isotropic molecular reorientation of a rigid sphere and, thus, a single correlation time from overall molecular reorientation (*τ*_mol_), then, the line width Δ*ν*_1/2_ can be expressed in the following form [11]:

(3) $$\Delta \nu_{1/2}=\frac{1}{\pi T_{2}}=\alpha_{0}^{}+\alpha_{1}\text{MW},$$

where MW is the molecular weight, *α*_1_ is the contribution to the line width of the quadrupolar coupling constant, density and temperature, (1), (2a), and (2b), and *α*_0_ is the solvent viscosity-independent contributions to the line width due to the primary hydration sphere of the amino acids. 

**Figure 4:**
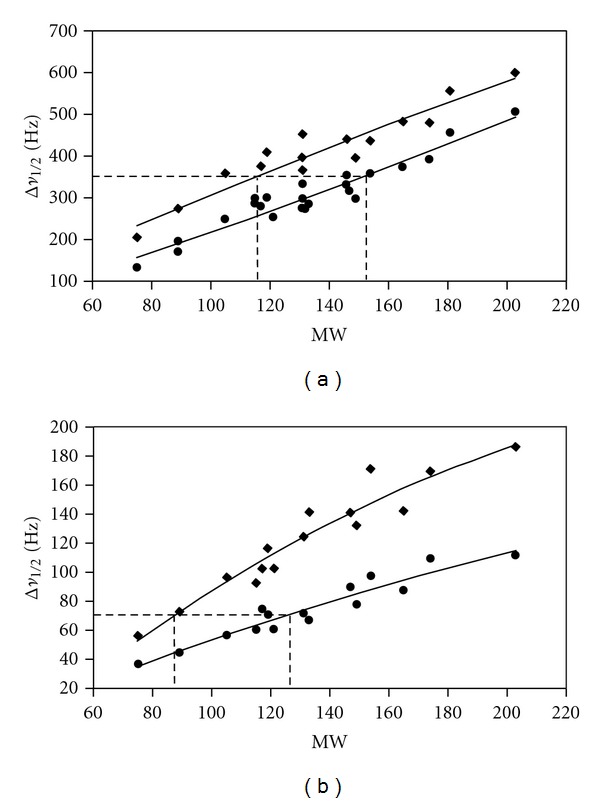
Plot of the ^14 ^N (28.9 MHz) (a) and ^17^O (48.8 MHz) (b) line widths, Δ*ν*_1/2_, of Table 1 of the protein amino acids versus their molecular weights, MW: (♦) pH 0.5, (•) pH 6.0. All lines correspond to a nonlinear least squares fit of the experimental points of Table 1 according to (7). Dotted lines indicate the difference in MW for the same Δ*ν*_1/2_ values. (Reprinted, with permission, from *J. Magn. Reson.* [11]. Copyright 2003, by Elsevier Inc.).

The linear correlation between Δ*ν*_1/2_ and MW at pH 6 for both ^14 ^N and ^17^O nuclei (Figure 4) is in agreement with the hydrodynamic model of (3) [11]. Furthermore, the *χ*(^17^O) of the amino acid is independent of both the ionization and the degree of hydration of the carboxyl group [10]. The increase in the ^17^O line widths at acidic pH (~100 ± 31 Hz), relative to those at neutral pH, was interpreted by a change in the rotational correlation time and, thus, effective MW of the amino acids, (3). This implies that the cationic form of the amino acids is more hydrated by an access of 1.3 to 2.5 molecules of water relative to that in the zwitterionic form [11] with lifetimes that are longer than the overall molecular rotational correlation time, presumably 2–10 ps [10].

In the case of a stochastic diffusion of the amino and carboxyl groups comprising contributions from internal (*τ*_int⁡_) and overall (*τ*_mol_) motions, the correlation time *τ*_*c*_ for ^14 ^N or ^17^O is given by [31]

(4a) $$\tau_{c}=\tau_{\text{mol}}\left[A+\left(B+C\right)\frac{\left(12/r\right)\tau_{\mathrm{int}}}{\tau_{\text{mol}}+\left(12/r\right)\tau_{\mathrm{int}}}\right]$$

with

(4b) $$A=\frac{3}{4}{\left(3\cos^{2}\theta -1\right)}^{2},\;\;B=3\sin^{2}\theta \cos^{2}\theta ,\;\;c=\frac{3}{4}\sin^{4}\theta ,$$

where *θ* is the angle between the rotation axis and the main field gradient (*r* denotes an *r*-fold jump mechanism). Since the sum of *A*, *B*, and *C* is equal to 1, (4a) can be rewritten as

(5a) $$\tau_{c}=\tau_{\text{mol}}\frac{A\tau_{\text{mol}}+\tau_{i}}{\tau_{\text{mol}}+\tau_{i}},$$

where

(5b) $$\tau_{i}\;=\;\left(\frac{12}{r}\right)\tau_{\mathrm{int}}.$$

Equations (5a) and (5b) can be rewritten as

(6) $$\tau_{c}=\left(1-A\right)\tau_{i}+A\tau_{\text{mol}}-\frac{\left(1-A\right)\tau_{i}^{2}}{\tau_{\text{mol}}+\tau_{i}}.$$

Since *A* and *τ*_*i*_ can be assumed to be constant for all the amino acids, (4a) and (4b) can be written as

(7) $$\Delta \nu_{1/2}=\alpha_{0}+\alpha_{1}\text{MW}+\frac{\alpha_{2}}{\text{MW}+\alpha_{3}},$$

where *α*_0_–*α*_3_ are constants. The minimization of (7) on the basis of the ^17^O experimental data gave the mean difference of 35.8 ± 17.3 in MW between pH 0.5 and 6.0 for three different Δ*ν*_1/2_ values: 250, 350 (Figure 4(b)), and 500 Hz. This was interpreted by an excess of 1–3 water molecules at pH = 0.5.

The difference in the ^14 ^N line widths at the two ionization states (Figure 4(a)) should be attributed to differences in the correlation times and to a decrease in the *χ*(^14^N) on deprotonation of the carboxyl group. In the case of the linear model, the influence of variations of values of the *χ*(^14^N) to the line width, Δ*ν*_1/2_, is less for small molecular weights. Therefore, for Δ*ν*_1/2_ = 70 Hz (Figure 4(a)), the difference in MW will be a reasonable approximation of the difference in hydration in the two states. The calculated value was found to be 45.2 ± 7.4, which corresponds to an excess of 2-3 water molecules in the cationic form compared to that in the zwitterionic form, in reasonable agreement with the ^17^O NMR data [11].

More recently, Takis et al. [32] investigated the C_*α*_^13^C longitudinal relaxation times (*T*_1_) and ^14 ^N line widths (Δ*ν*_1/2_) of amino acids and acetyl-amino acids in aqueous solutions at acid and neutral pH. Both ^13^C_*α*_ and ^14 ^N values indicate that amino acids and acetyl-amino acids at acid pH interact with an access of one water molecule with respect to their deprotonated form at neutral pH. On the contrary, ^13^C_*α*_ and ^14 ^N values of betaines (R_3_N^+^CH(R)COO^−^) demonstrate no hydration differences in acid and neutral pH values.

### 2.3. Crystallographic Data and Statistics

Crystal structure databases provide a rich source of information to extract details on the architectures and interactions of molecules. This kind of search provides the opportunity to examine the formation of intramolecular and intermolecular hydrogen bond in small molecule crystal structures [33, 34]. Propensities for the hydration of the *α*-carboxylate group of amino acids and their derivatives were derived on the basis of exhaustive searches in the Cambridge Crystallographic Database (CSD). Since intermolecular hydrogen bonds are preferred when five- or six-membered conjugated rings are formed [35], particular attention has been given to the hydrogen bond patterns in the vicinity of the carboxylate group that involves two simultaneous hydrogen acceptors. The concept of five- and six-membered conjugated rings, along with three-center (bifurcated) and 4-center (trifurcated) hydrogen bonds, has been acknowledged and accepted widely as an important factor in determining the structure and function of molecules ranging from inorganic to organic and biological molecules [1, 35–39]. Furthermore, Port and Pullman [40] studied theoretically the formate ion-water interaction as a prototype of the carboxylate group. Three energetically favourable hydration sites were obtained, two equivalent sites on the carboxylate oxygens at the exterior of the ion and one water bridging the two oxygen atoms.

The ConQuest 1.13 program was used for all the statistical analysis described in this paper. Specifically, the CSD version 5.32 (November 2010) for small molecules was searched, with the following general search flags: *R* > 0.5, “3D coordinates”, and “only organic”.

**Figure 5:**
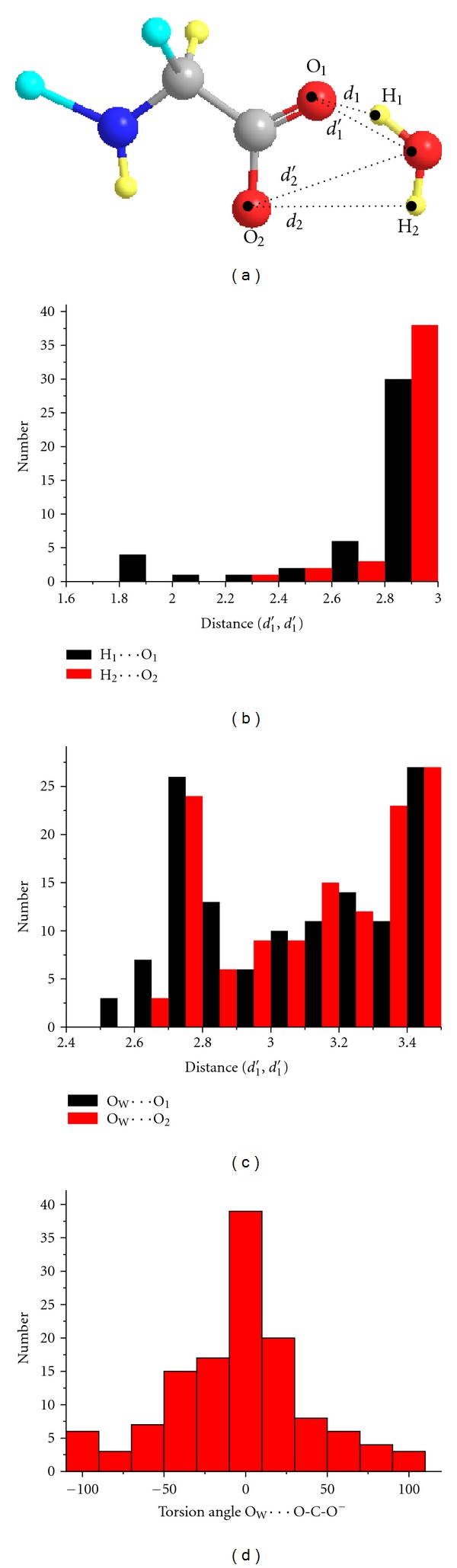
(a) Cambridge Crystallographic Database (CSD) search queries for six-membered hydrogen bond conjugated rings between the two oxygens of the *α*-carboxylate with a molecule of water in the vicinity. Hydrogen bonds and long-range dipolar interactions are defined by the geometric constraints *d*_1_ and *d*_2_ ≤ 3 Å and *d*_1_′ and *d*_2_′ ≤ 3.5 Å. C (gray), N (blue), any group (cyan), H (light-yellow), and O (red). (b) Plot of the number of structures *versus* the distances *d*_1_(H_1_ *⋯*O_1_) and *d*_2_(H_2_ *⋯*O_2_). (c) Plot of the number of structures *versus* the distances *d*_1_′**(**O_W_ *⋯*O_1_) and *d*_2_′(O_W_ *⋯*O_2_). (d) Distribution of the torsion angle O_1_–C–O_2_ *⋯*O_W_ *versus* the number of structures.

In order to extract the number of entries present in the current database that form six-membered conjugated rings between the two oxygens of the *α*-carboxylate and the carboxylic group with a molecule of water in the vicinity, the following geometric cut-offs were used: upper limits *d* = 3 Å for (O_w_)–H*⋯*O=C and (O_w_)–H⋯^−^O–C, and *d*′ = 3.5 Å for O_w_ *⋯*O=C and O_w_ *⋯* ^−^O–C. 44 hits were obtained for the carboxylate state (Figure 5), whereas only one was derived for the protonated form. Therefore, the number of structures of carboxylates is sufficient to give reasonable statistics. Figure 5 demonstrates that the oxygen of water, O_w_, is reasonably close to the carboxylate oxygens and displays a significant preference for the O_1_–C–O_2_ carboxylate plane.

**Figure 6:**
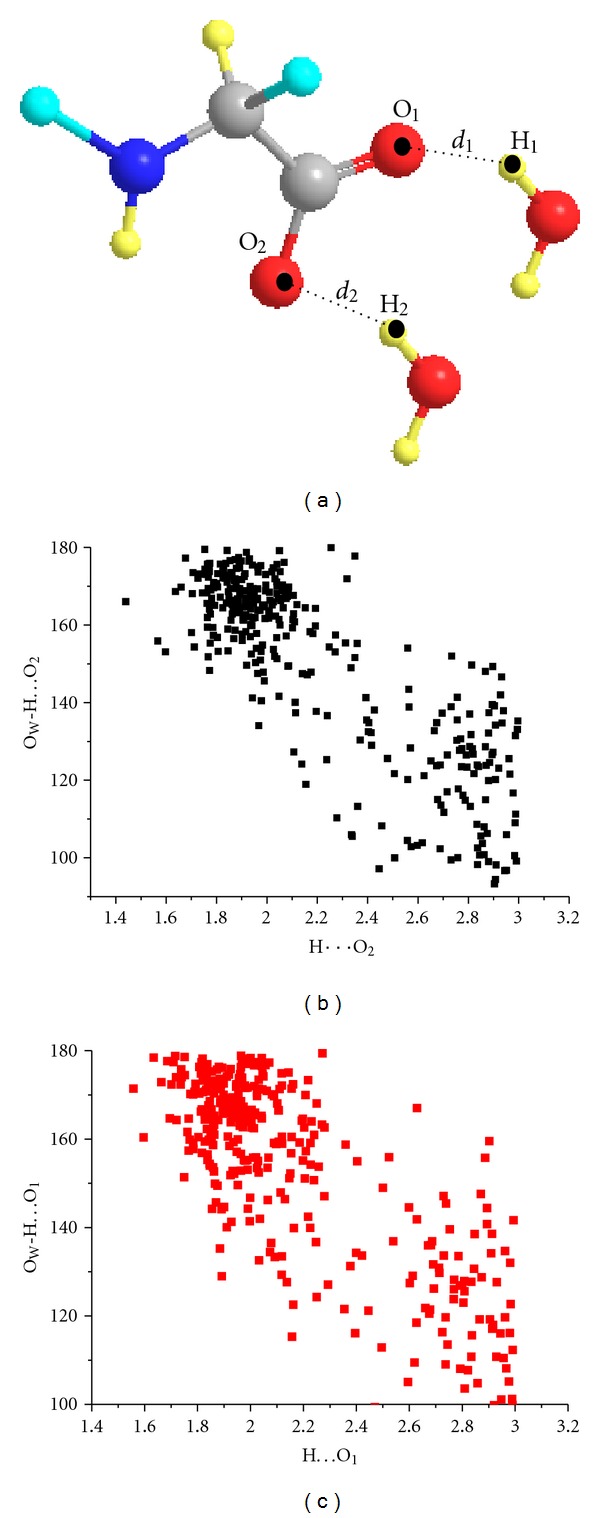
Cambridge Crystallographic Database (CSD) search query for the structural mode (a) C (gray), N (blue), any group (cyan), H (light-yellow), and O (red) of a bound water molecule to carboxylates. Correlations between O_W_–H*⋯*O_2_ distances and O_W_–H*⋯*O_2_ angles (b) and O_W_–H*⋯*O_1_ distances and O_W_–H*⋯*O_1_ angles (c).

There is a general correlation between hydrogen bond lengths and hydrogen bond angles (Figure 6) similar to that observed by Jeffrey and Maluszynska [41] in the case of water molecules in the hydrates of small biological molecules. Shortening of the O_W_–H*⋯*O_2_ and O_W_–H*⋯*O_1_ distances implies larger O_W_–H*⋯*O_1_ and O_W_–H*⋯*O_2_ angles.

**Figure 7:**
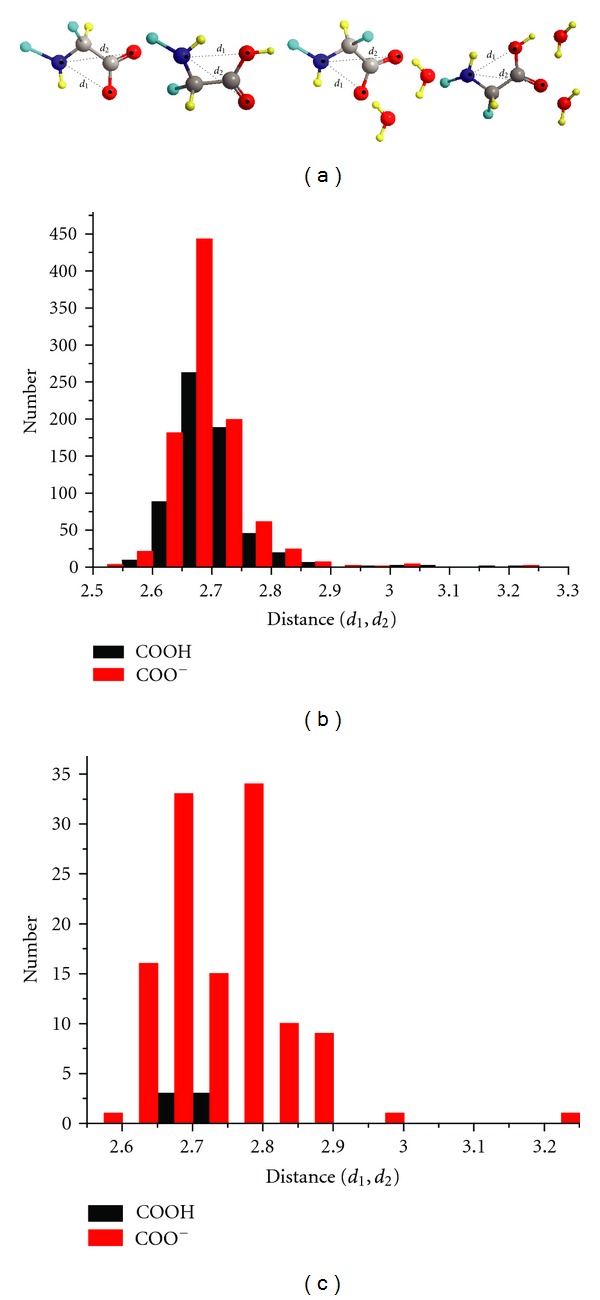
(a) Cambridge Crystallographic Database (CSD) search queries for intramolecular hydrogen bond in the presence and in the absence of two water molecules bound to the carboxylate and carboxylic groups, respectively. C (gray), N (blue), any group (cyan), H (light-yellow), and O (red). (b) Plot of the number of structures *versus* the distances *d*_1_(N*⋯*O^−^) and *d*_2_(N*⋯*O=C) in the absence of a water molecule. (c) Plot of the number of structures *versus *the distances *d*_1_(N*⋯*O^−^) and *d*_2_(N*⋯*O=C) in the presence of two water molecules bound to the carboxylate and the carboxylic groups, respectively.

Furthermore, crystallographic database searches were performed to identify the propensity for the formation of intramolecular hydrogen bond interaction in the carboxylate (NH*⋯*O^−^) and the carboxylic acid (NH*⋯*O=C) state. Interestingly, 946 and 118 hits were retrieved for the carboxylate and 621 and 6 hits for the carboxylic form in the absence and presence of two molecules of water, respectively. It is evident from Figure 7 that in the presence of two bound water molecules there is a significant reduction in the number of structures with intramolecular hydrogen bond interaction for the carboxylate group and, concurrently, a significant increase in the distance (NH*⋯*O^−^). It is important to note that no intramolecular ^+^NH_3_ *⋯* ^−^OOC hydrogen bonds were observed for 82 amino acid carboxylates with sp^3^-hybridized C^*β*^-atoms [42] in agreement with an early survey of amino acid structures determined by neutron diffraction [43].

## 3. Conclusions

**Figure 8:**
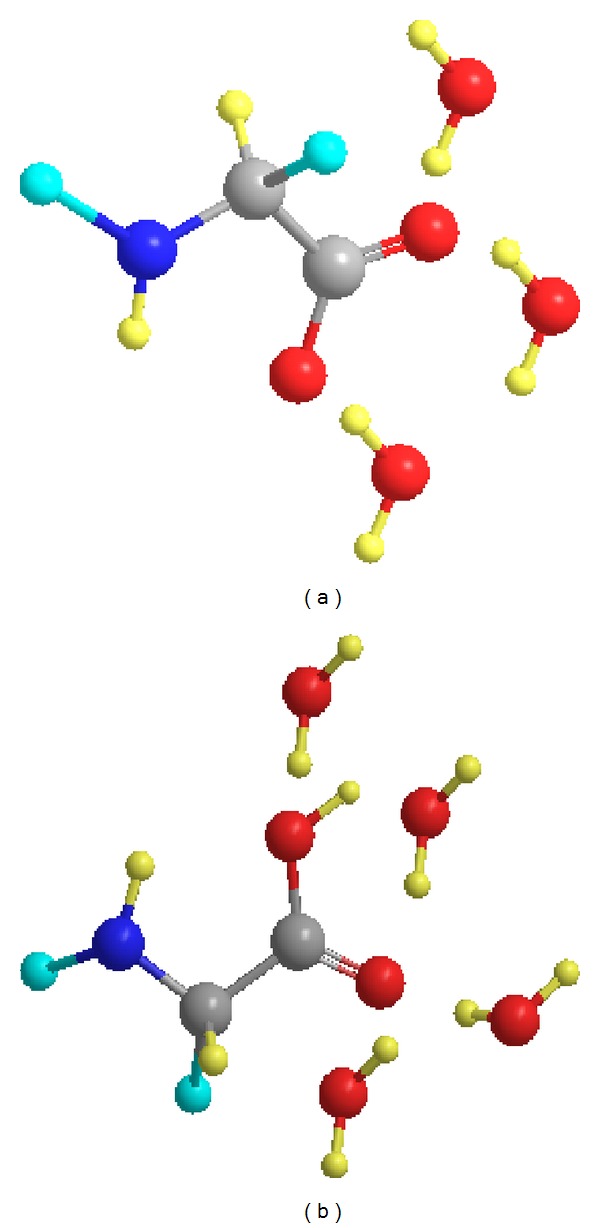
Tentative models for the hydration sites of the *α*-carboxylic (a) and carboxylate (b) group of amino acids and their derivatives. C (gray), N (blue), any group (cyan), H (light-yellow), and O (red).

^17^O shielding changes of amino acids as a function of molar fraction of DMSO/H_2_O, the decrease in the longitudinal relaxation times (*T*_1_) of C_*α*_ D and ^13^C_*α*_, and the increase in line widths of ^14 ^N and ^17^O at acidic pH relative to those at neutral pH may be interpreted with the hypothesis that the cationic form of amino acids is more hydrated by 1 to 3 molecules of water than the zwitterionic form. Similar behaviour was also observed for acetylated derivatives of amino acids, but not for betaines, between the protonated and deprotonated carboxyl group. Although the precise hydration differences observed for various nuclei deviate somehow, it may be concluded that these hydrated complexes have lifetimes that are shorter than the NMR chemical shift time scale, but presumably longer than the overall molecular rotational correlation time of 2–10 ps. An exhaustive search in the Cambridge Crystallographic Database (CSD) demonstrates a strong tendency of the two oxygens of the deprotonated carboxylate group to form hydrogen bonds with a single molecule of water. Even though statistical analysis of structural parameters in crystals cannot be used in a straightforward way to derive quantitative structural models in solution, it is of interest to note that this mode of six-membered conjugated ring, which is absent in the case of the carboxylic group, might result in a more compact and, thus, less hydrated structure in aqueous solution, in accordance with the NMR data (Figure 8).

Furthermore, it may be concluded that the bound molecules of water alleviate the NH*⋯*O^−^ interaction and very probably this effect is even more pronounced in aqueous solution. From the above, it is evident that the reduced hydration of the carboxylate group, relative to the carboxylic group, should be attributed mainly to the strong tendency of the carboxylate group to form a six-membered conjugated ring with a single molecule of water. The NH*⋯*O^−^ intramolecular hydrogen bond very probably plays an insignificant role. Constructively, the tentative models illustrated in Figure 8 should be further validated by *in silico* and experimental approaches. Computational methods complement the experimental results by providing information on the microscope and physicochemical details on the interplay between water and the biomolecule of interest [44–47]. For example, introduction of solvent effects into molecular dynamics can provide an atomic description of the folding and unfolding of a protein [47]. Furthermore, there is an array of theoretical approaches that have been utilized for treating NMR shieldings in solution [48], that can be classified as continuum models [49, 50] and molecular dynamics simulations [51]. Experimental approaches could involve ^17^O NMR both in powders and in the crystal state [52] with varying degrees of hydration.

## Figures and Tables

**Table 1:** ^14 ^N (at 28.9 MHz) and ^17^O (at 48.8 MHz) linewidths, Δ*ν*_1/2_, of protein amino acids in different ionization states^∗a^.

Amino Acid	MW^f^	Δ*ν*_1/2_, Hz^b,c^			
		pH 0.5		pH 6.0	
		^14^N	^17^O	^14^N	^17^O
Gly	75.07	55 (67)	205 (233)	36 (40)	132 (131)
Ala	89.10	71 (79)	276 (274)	45 (49)	194 (172)
Sar	89.10	83 (80)	272 (274)	60 (49)	170 (172)
N,N-dimethyl-Gly	103.10		329 (314)		198 (212)
Ser	105.09	97 (94)	359 (320)	57 (58)	249 (218)
Pro	115.13	87 (104)	286 (349)	60 (64)	197 (247)
Val	117.15	102 (105)	376 (355)	72 (65)	280 (253)
Thr	119.12	118 (107)	410 (360)	71 (66)	300 (259)
Cys	121.26	104 (109)	e	59 (67)	254 (265)
4-hydroxy-Pro	131.13		366 (395)		275 (293)
Ile	131.18	115 (118)	453 (395)	65 (73)	335 (294)
Leu	131.18	124 (118)	397 (395)	71 (72)	298 (294)
Asn	132.12	d	e	d	274 (296)
Asp	133.11	140 (120)	d	65 (74)	285 (299)
Gln	146.15	d	e	d	330 (337)
Lys	146.19	d	443 (439)	d	354 (337)
Glu	147.13	146 (133)	d	89 (82)	316 (340)
Met	149.21	133 (135)	397 (448)	78 (83)	297 (346)
His	155.16	169 (140)	438 (465)	99 (86)	359 (363)
Phe	165.19	143 (150)	485 (494)	88 (93)	376 (392)
Arg	174.20	165 (159)	482 (520)	111 (98)	394 (418)
Tyr	181.19	134(164)	558 (540)	102 (102)	457 (438)
O-Methyl-Tyr	195.19		622 (581)		525 (479)
Trp	204.23	180 (186)	602 (607)	108 (115)	508 (505)

*Adapted, with permission, from J. Magn. Reson. [11]. Copyright 2003, by Elsevier Inc.

^a^0.1 M solutions in H_2_O containing 10^-5 ^M EDTA (ethylenediamine-N,N,N′,*Ν*′-tetraacetate); *T* = 40°C.

^b ^Linewidths at half-height, estimated errors <±5%.

^c^Values into parenthesis correspond to line widths resulting from regression analysis assuming isotropic molecular motion and linear approximation.

^d^Overlapping resonances.

^e^Not measured because of degradation.

^f^MW of the zwitterionic form.
